# Exploring the coordination chemistry of ruthenium complexes with lysozymes: structural and in-solution studies

**DOI:** 10.3389/fchem.2024.1371637

**Published:** 2024-04-04

**Authors:** Maria Oszajca, Monika Flejszar, Arkadiusz Szura, Patrycja Dróżdż, Małgorzata Brindell, Katarzyna Kurpiewska

**Affiliations:** ^1^ Department of Inorganic Chemistry, Faculty of Chemistry, Jagiellonian University, Kraków, Poland; ^2^ Department of Physical Chemistry, Faculty of Chemistry, Rzeszów University of Technology, Rzeszów, Poland; ^3^ Department of Crystal Chemistry and Crystal Physics, Faculty of Chemistry, Jagiellonian University, Kraków, Poland

**Keywords:** lysozyme, ruthenium complexes, crystal structure, adduct, metallodrugs, protein–ligand complex

## Abstract

This study presents a comprehensive structural analysis of the adducts formed upon the reaction of two Ru(III) complexes [HIsq][*trans*-Ru^III^Cl_4_(dmso)(Isq)] (**1**) and [H_2_Ind][*trans*-Ru^III^Cl_4_(dmso)(HInd)] (**2**) (where HInd–indazole, Isq–isoquinoline, analogs of NAMI-A) and two Ru(II) complexes, *cis*-[RuCl_2_(dmso)_4_] (**c**) and *trans*-[RuCl_2_(dmso)_4_] (**t**), with hen-egg white lysozyme (HEWL). Additionally, the crystal structure of an adduct of human lysozyme (HL) with ruthenium complex, [H_2_Ind][*trans*-RuCl_4_(dmso)(HInd)] was solved. X-ray crystallographic data analysis revealed that all studied Ru complexes, regardless of coordination surroundings and metal center charge, coordinate to the same amino acids (His15, Arg14, and Asp101) of HEWL, losing most of their original ligands. In the case of the **2**-HL adduct, two distinct metalation sites: (i) Arg107, Arg113 and (ii) Gln127, Gln129, were identified. Crystallographic data were supported by studies of the interaction of **1** and **2** with HEWL in an aqueous solution. Hydrolytic stability studies revealed that both complexes **1** and **2** liberate the N-heterocyclic ligand under crystallization-like conditions (pH 4.5) as well as under physiological pH conditions, and this process is not significantly affected by the presence of HEWL. A comparative examination of nine crystal structures of Ru complexes with lysozyme, obtained through soaking and co-crystallization experiments, together with in-solution studies of the interaction between **1** and **2** with HEWL, indicates that the hydrolytic release of the N-heterocyclic ligand is one of the critical factors in the interaction between Ru complexes and lysozyme. This understanding is crucial in shedding light on the tendency of Ru complexes to target diverse metalation sites during the formation and in the final forms of the adducts with proteins.

## 1 Introduction

**FIGURE 1:**
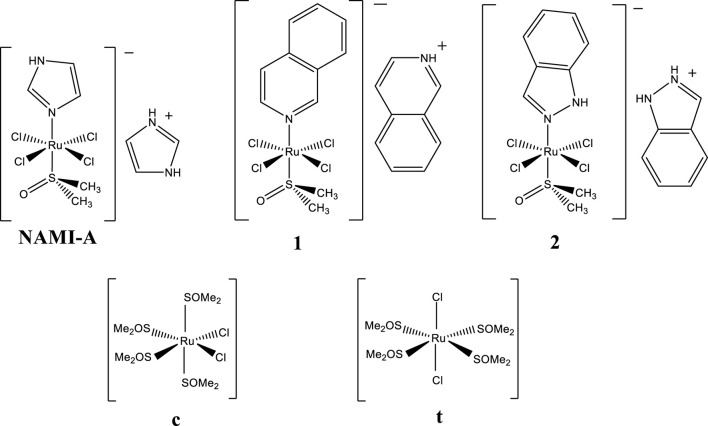
NAMI-A (H_2_Im)[*trans*-RuCl_4_(dmso)(HIm)] and the complexes studied in this work: [HIsq][*trans*-RuCl_4_(dmso)(Isq)] (**1**), [H_2_Ind][*trans*-RuCl_4_(dmso)(HInd)] (**2**), *cis*-[RuCl_2_(dmso)_4_] (**c**), and *trans*-[RuCl_2_(dmso)_4_] (**t**) where HIm–imidazole, Isq–isoquinoline, HInd–indazole, and dmso–dimethyl sulfoxide (SOMe_2_).

New anti-metastasis inhibitor, NAMI-A, [H_2_Im][*trans*-RuCl_4_(dmso)(HIm)] (Figure 1), is a ruthenium complex that, due to its initially very promising antimetastatic properties, has attracted the attention of numerous scientists and designated a new direction in research on metal complexes as potential anticancer drugs (Keppler et al., 1987; Alessio et al., 1993; Mestroni et al., 1994; Lipponer et al., 1996; Bergamo et al., 2002; Enzo; Alessio et al., 2005; Levina et al., 2009; Ang et al., 2011; Komeda and Casini, 2012; Alessio, 2017; Riccardi et al., 2017). NAMI-A successfully passed phase I of clinical trials (Rademaker-Lakhai et al., 2004; Liu et al., 2013) and is the first ruthenium complex to reach phase II (Leijen et al., 2015). Despite the great number of preclinical studies demonstrating extraordinary properties of NAMI-A (Bergamo and Sava, 2015), the clinical development of NAMI-A seems to have come to a standstill due to the results of the clinical trials phase I/II not meeting expectations in patients with non-small-cell lung cancer treated with a gemcitabine and NAMI-A combination (Leijen et al., 2015). One of the reasons for such a failure may have been an inadequate therapeutic procedure that considered NAMI-A as a typical cytotoxic agent like cisplatin rather than a metastasis inhibitor. Promising preclinical data encourage researchers to develop better analogs of NAMI-A.

This article presents studies on [HIsq][*trans*-RuCl_4_(dmso)(Isq)] (**1**) and [H_2_Ind][*trans*-RuCl_4_(dmso)(HInd)] (**2**) that belong to a large family of NAMI-A analogs (Figure 1). They differ from NAMI-A only by the N-heterocyclic ligand coordinated in the *trans* position with respect to the dmso ligand, as well as with the counterion (Figure 1). Previous studies showed that the hydrolytic stability of these complexes under physiological conditions is comparable to NAMI-A, with one distinct difference being that, unlike NAMI-A, they release their N-heterocyclic ligand (Oszajca et al., 2017). Biological studies revealed interesting antiangiogenic properties of **1** and **2** under biologically relevant hypoxic conditions. Among the three complexes, **1**, **2**, and NAMI-A, **1** exhibited the most efficient hypoxia- and dose-dependent inhibition of angiogenesis when the compounds were evaluated for their influence on pseudo-vessels formation by microvascular endothelial cells (HSkMEC) (Oszajca et al., 2016).

The mechanism of action of NAMI-A-type ruthenium complexes is still not completely understood, and their anticancer properties, in contrast to platinum-based complexes targeting DNA, seem to be tuned by the formation of adducts with proteins. It is commonly believed that the interaction of ruthenium complexes with proteins plays a crucial role in the complexes’ toxicity, biodistribution, bioavailability, and mechanism of action (Anthony et al., 2020; Frei et al., 2020; Xu et al., 2023). X-ray crystallographic studies provided evidence that NAMI-A (Chiniadis et al., 2021), as well as its analog (AziRu) with a pyridine ligand instead of imidazole, forms adducts with model proteins (Casini et al., 2010; Vergara et al., 2013b; Messori and Merlino, 2014). Interestingly, both ruthenium complexes lose all their ligands upon protein binding, resulting in a complete change of the coordination environment of the ruthenium center. The analysis of NAMI-A and AziRu adducts with HEWL showed that, although these two complexes have highly similar structures, the adducts they form with proteins vary significantly in their metalation sites. This intriguing finding suggests that the characteristics of the coordinated ligands, which are absent in the final structures, can play a crucial role in determining the protein-binding site. It appears that N-heterocyclic ligands are involved in non-covalent interactions during the initial phase of the binding process (Vergara et al., 2013b; Messori and Merlino, 2014). Similar results have been reported for the interaction of indazolium *trans*-[tetrachlorobis (1H-indazole)ruthenate(III)] (KP1019) and its analog sodium *trans*-[tetrachlorobis(1H-indazole)ruthenate(III)] (NKP-1339) with albumin. The combined findings from structural analysis and inductively coupled plasma mass spectrometry (ICP-MS) indicate that two “naked” ruthenium ions coordinate with histidine residues situated in the hydrophobic binding pockets of albumin (Bijelic et al., 2016). Although the liberation of both indazole ligands from the coordination sphere was confirmed, their important role in the recognition of the binding site seems to be highly probable.

Currently, scientists are increasingly focused on gaining a more detailed understanding of how metallodrugs interact with proteins at the molecular level, as this knowledge is crucial in defining the pharmacological functions of these drugs (Anthony et al., 2020; Frei et al., 2020; Xu et al., 2023). The formation of adducts between ruthenium and proteins, for instance, can modify the catalytic efficiency of various enzymes (Bergamo and Sava, 2007). These changes may play a crucial role in determining the manner in which such complexes express their biological activities, whether in combating cancer cells (Nyong-Bassey et al., 2023) or in addressing neurodegeneration in Alzheimer’s disease (Singh et al., 2023).

In view of these recent findings, we have conducted detailed studies on the reaction of **1** and **2** with HEWL as the model protein. In our study, we chose two NAMI-A-type ruthenium complexes in order to examine whether the nature of the N-heterocyclic ligand and the hydrolytic behavior of the studied complexes affect modes of the metallodrug–protein interaction. The crystal structure analysis of the adducts formed by **1** and **2** with HEWL and by **2** with HL is also reported here, which is the first structure of the ruthenium complex determined with human lysozyme. Crystallographic studies were also performed for adducts formed by HEWL with *trans*-[RuCl_2_(dmso)_4_] (**t**) and *cis*-[RuCl_2_(dmso)_4_] (**c**) (Figure 1) to better understand the influence of the metal oxidation state and the coordination surroundings of the ruthenium center on the selection of binding site and degree of occupancy. These studies were supplemented with solution-based determination of Ru fraction bound to HEWL upon interaction with **1** and **2**, as well as the affinity of both complexes to this protein. Furthermore, the hydrolytic stability of **1** and **2** and the influence of HEWL on the degradation processes were investigated. The crystallographic data of several Ru-protein adducts presented in this study, together with the interaction of Ru complexes with lysozyme studied in solution, allowed us to draw conclusions about the factors governing the selection of metalation sites by NAMI-A-type complexes. Our results nicely supplement the studies reported so far on the interaction of anticancer ruthenium compounds with proteins (Casini et al., 2010; Vergara et al., 2013b; Messori and Merlino, 2014; Bijelic et al., 2016; Merlino, 2016), providing evidence for additional crucial factors that cannot be omitted when discussing ruthenium metalation of proteins by labile ruthenium complexes.

## 2 Materials and methods

### 2.1 Materials and reagents

All chemicals used in this study were of analytical reagent grade. Ruthenium complexes [HIsq][*trans*-RuCl_4_(dmso)(Isq)] (**1**), [H_2_Ind][*trans*-RuCl_4_(dmso)(HInd)] (**2**), *cis*-[RuCl_2_(dmso)_4_] (**c**), and *trans*-[RuCl_2_(dmso)_4_] (**t**) where HInd–indazole, Isq–isoquinoline, and dmso–dimethyl sulfoxide were prepared following the published procedure (Mestroni et al., 1988; Zorzet et al., 2001; Reisner et al., 2004; Cardoso et al., 2014). Purity was confirmed by elemental analysis. Lysozyme from chicken egg white (HEWL) (≥40,000 units/mg protein), human lysozyme (HL) (≥100,000 units/mg protein), ammonium sulfate, PEG4000, sodium acetate (>99%), Tris buffer (2-amino-2-(hydroxymethyl)-1,3-propanediol, >99.8%), Dowex^®^ Marathon™ C sodium form, and NaCl were purchased from Sigma-Aldrich.

### 2.2 Synthesis of Na[*trans*-RuCl_4_(dmso)(Isq)] (1) and Na[*trans*-RuCl_4_(dmso)(HInd)] (2)

Ruthenium complexes **1** and **2** were dissolved in 0.1 M HCl and stirred with preconditioned (30 min) Dowex^®^ Marathon™ C resins (2 g/4 mL) for 30 min. Subsequently, the resin was filtered off, and the procedure was repeated once for **1** and twice for **2** with a fresh portion of Dowex^®^ Marathon™ C resins. The effectiveness of ion exchange was confirmed by fluorescence measurements because both coordinated N-heterocyclic ligand molecules acting as counter ions and HIsq^+^ and H_2_Ind^+^ exhibit emission spectra (Supplementary Figure S1). The stability of ruthenium complexes during the ion-exchange procedure was confirmed spectrophotometrically and by high-performance liquid chromatography (HPLC).

### 2.3 Protein crystallography

Crystals of HEWL with ruthenium complexes were grown at room temperature using the hanging drop vapor diffusion method according to Weiss et al. (2000). A solution of 20 mg/mL HEWL was co-crystallized with **1** and **2**, all dissolved in water. The drop was composed of 1.5 μL of the protein, 0.5 μL of 4 mM [H_2_Ind][*trans*-RuCl_4_(dmso)(HInd)] and 14 mM [HIsq][*trans*-RuCl_4_(dmso)(Isq)], and 2 μL of the reservoir solution. The crystallization solution was composed of an NaCl (0.8 M) acetate buffer (0.05 M, pH 4.5). Similarly, a 20 mg/mL HEWL solution was used for crystallization and co-crystallization with **c** and **t** (all solved in water). The crystallization solution contained NaCl (1 M) and acetate buffer (0.05 M, pH 4.5). The drop was composed of 1.5 μL of the protein, 2 μL of reservoir solution, and 0.5 μL of **1** or **2** (4 mM) or 0.5 μL of **c** or **t** (10 mM), respectively. Drops were equilibrated against 0.5 mL of reservoir solution. Brown, green, yellow, and orange crystals appeared after 2 weeks of HEWL co-crystallization with **1**, **2**, **c**, and **t**, respectively. Crystal soaking was conducted by adding 0.5 μL of **1**, **2** (4 mM) or **c**, **t** (4 mM) to the drop. The crystals exhibited coloration in a few hours. Comparable color alterations were noted over time for the ligand solutions. Interestingly, even though the co-crystallization and soaking experiments resulted in colored crystals, not all of them proved to contain a ligand bound to HEWL, which suggests that the crystals might also contain deeply colored polynuclear Ru species that resulted from the prolonged hydrolysis.

The lyophilized HL was dissolved in MiliQ water to a final concentration of 6 mg/ml. A drop consisting of 1.5 μL of protein solution, 0.5 μL of 4 mM of **2** (dissolved in water), and 2 μL of the reservoir solution was equilibrated against 0.5 mL of reservoir solution containing (NH_4_)_2_SO_4_ (0.2M), CH_3_COONa (0.1M), and PEG4000 (21%). Yellow crystals appeared after a week. Crystals of HL were not obtained in the presence of **1**, **c**, or **t**, either through co-crystallization or soaking. The cryoprotected crystals (30% glycerol in crystallization solution) were flash-cooled in liquid nitrogen, and data for co-crystallized HEWL with **1**, **2**, **c**, and **t** and HL with **2** were collected using a SuperNova (*Rigaku Oxford Diffraction*) four-circle diffractometer with a mirror monochromator and a microfocus CuKα radiation source (λ = 1.5418 Å). Diffraction images of HEWL crystals soaked with **1**, **2**, **c**, and **t** for 5 h were collected at the 14.1 beamline at the BESSY II electron storage ring operated by the Helmholtz-Zentrum Berlin für Materialien und Energie. Data were collected at 100 K using a wavelength of 0.9184 Å. The MXCuBE (Gabadinho et al., 2010) user interface was used for data collection, and XDSAPP was used for auto-processing (Krug et al., 2012). Structures were solved using the molecular replacement method (*PHASER*) (McCoy et al., 2007) using the coordinates of HEWL (PDBid: 1H87) (Girard et al., 2002) and HL (PDBid: 3LN2) (Gill et al., 2011) as starting models. The selection of the high-resolution cutoff for data processing was based on the value of <I/σ(I)> (data cutoff at 2.0). Crystallographic refinement was carried out using maximum-likelihood target functions implemented in *Refmac5* (Potterton et al., 2004). The final models, including refinement of Ru occupancy, were automatically performed by *Phenix* (Adams et al., 2010). Each round of refinement was supplemented with a round of model building using *WinCOOT* (Emsley and Cowtan, 2004). A test dataset to monitor R_free_ was established using 5%–10% of randomly selected data. As the phases improved, metal ions and ordered solvent molecules were added manually. Finally, the structures were validated using *MolProbity* (Chen et al., 2010). Structures resulting from co-crystallization were submitted to the Protein Data Bank (Berman et al., 2000) with the following codes: 5LVG (**c**-co-HEWL), 5LVH (**t**-co-HEWL), 5LVI (**1**-co-HEWL), 5LVJ (**2**-co-HEWL), and 5LVK (HL with **2**). Structures resulting from the soaking experiments were submitted with the following codes: 8RNV (**c**-so-HEWL), 8RNW (**t**-so-HEWL), 8RNX (**1**-so-HEWL), and 8RNY (**2**-so-HEWL).

### 2.4 Ruthenium–lysozyme adduct preparation

An aliquot of **1** or **2** stock solution was mixed with a buffered solution of HEWL in acetate (0.05 M, pH 4.5, 0.2 M NaCl) or Tris (0.1 M, pH 7.4, 0.2 M NaCl) buffer to give a 20-fold molar excess of the Ru complex over the protein in the final solution, with a 1.4 × 10^−4^ M concentration of protein. The resulting mixture was incubated for 24 h at 37°C, then filtered to remove any precipitations arising from the formation of insoluble polynuclear Ru species, followed by ultrafiltration through a 3 kDa cutoff filter for 10 min (37°C, 13.3G) to remove free Ru species (unbound to protein). The ultrafiltration was repeated three times; each time, the protein was dissolved with a fresh portion of an appropriate buffer. After reverse ultrafiltration, the adduct solution was mixed with 500 μL of appropriate buffer. The final protein concentration in each sample was measured using a Bradford assay. The total ruthenium content in the samples was measured by application of ICP-MS using an ELAN 6100 PerkinElmer spectrometer. Prior to the determination of Ru content, 100 μL of the samples was mineralized using 500 μL of ultrapure concentrated nitric acid and then diluted with water.

### 2.5 Spectrofluorimetric and spectroscopic measurements

Fluorescence spectra were recorded on a PerkinElmer LS55 spectrofluorimeter equipped with a thermostat (Grant LTD6G) (±0.1°C) in a 1-cm quartz cuvette at 37°C. In a typical fluorescence quenching experiment, an aqueous solution of ruthenium complex **1** or **2** was added to the HEWL solution either in acetate buffer (0.05 M, pH 4.5, 0.2 M NaCl) or in Tris buffer (0.1 M, pH 7.4, 0.2 M NaCl). The samples always contained 2 µM HEWL, and various HEWL–ruthenium complex (**1**, **2**) ratios were used. The emission spectra were recorded in the wavelength range of 305–500 nm upon excitation at 295 nm. The fluorescence spectra were corrected for self-absorbance and inner-filter effects according to the equation: $F_{corr}=F_{obs}\times 10^{\frac{A_{ex}+A_{em}}{2}}$ , where *F*_*corr*_ and *F*_*obs*_ are the corrected and observed fluorescence intensity values, respectively, and *A*_*ex*_ and *A*_*em*_ are the absorbance values at the excitation and emission wavelengths, respectively (Instrumentation for Fluorescence Spectroscopy, 2006). UV-Vis absorption spectra were recorded on a PerkinElmer Lambda 35 UV-Vis spectrophotometer.

### 2.6 HPLC measurements

The chromatograms were registered with a PerkinElmer HPLC Chromera system equipped with a diode-array and a fluorescence detector. A Brownlee^TM^ Bio C18 column with particle size 5 µm, pore size 300 Å, 150 × 4.6 mm was used for the HPLC separation. Separation methods for quantification of N-heterocyclic ligand release: 0.05 M acetate buffer pH 4.5/CH_3_CN – 90/10 for 1 min, gradient to 50/50 for 15 min, 4 min hold; flow rate 1 mL/min. Chromatograms were recorded at excitation λ = 295 nm and emission λ = 350 nm. All the solutions used in the experiments were prepared in deionized water.

## 3 Results

### 3.1 Overall structures of HEWL and HL adducts with Ru complexes

**FIGURE 2:**
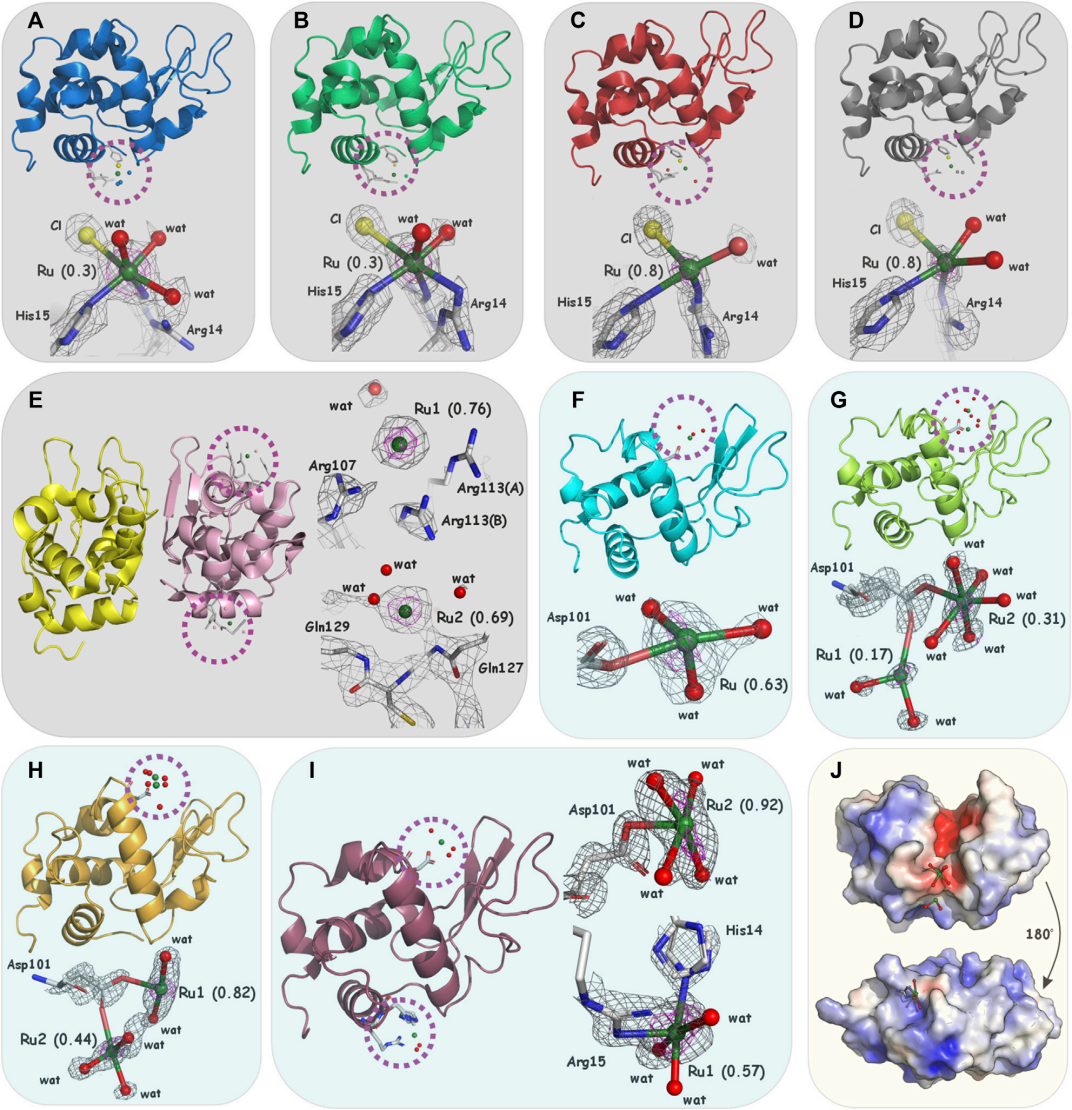
Overall structure and details of metalation sites in **(A) 1**-co-HEWL (PDBid: 5LVI, blue), **(B) 2**-co-HEWL (PDBid: 5LVJ, green), **(C) c**-co-HEWL (PDBid: 5LVG, red), **(D) t**-co-HEWL (PDBid: 5LVH, gray), **(E) 2**-HL (PDBid: 5LVK, yellow/pink), **(F) 1**-so-HEWL (PDBid: 8RNX, cyan), **(G) 2**-co-HEWL (PDBid: 8RNY, lemon), **(H) c**-co-HEWL (PDBid: 8RNV, orange), and **(I) t**-co-HEWL (PDBid: 8RNW, violet). For **(A–E)** (light gray boxes), the *2F*_*o*_ *-F*_*c*_ electron density map is contoured at 1.0–1.5*σ* (gray) and 2.0–3.0*σ* (magenta), for **(F–I)** (light blue boxes), the *2F*_*o*_ *-F*_*c*_ electron density maps are contoured at 1.0–1.5*σ* (gray) and anomalous scattering density at 3.0*σ* (magenta). **(J)** The surface is color-coded according to the electrostatic potential with negative values at −4kT/e in red, neutral in white, and positive at +4kT/e in blue; upper Ru1 next to Asp101 in **2**-co-HEWL, lower Ru1 next to His14 in **t**-co-HEWL (light yellow box). Atom colors for the complexes are white for C, blue for N, red for O, yellow for Cl, and green for Ru.

Single crystals of lysozyme (HEWL and HL) with the studied ruthenium complexes were obtained by co-crystallization (-co-) or soaking (-so-). All HEWL ruthenium adducts belonged to the P4_3_2_1_2 space group and, in the case of **2**-HL, to P2_1_2_1_2_1_. Crystals of **1**-co-HEWL, **2**-co-HEWL, **c**-co-HEWL, **t**-co-HEWL, and **2**-co-HL were solved to maximum resolutions of 1.70 Å, 1.60 Å, 2.0 Å, 1.55 Å, and 2.49 Å, respectively. Crystals of **1**-so-HEWL, **2**-so-HEWL, **c**-so-HEWL, and **t**-so-HEWL were solved to maximum resolutions of 1.25 Å, 1.02 Å, 1.08 Å, and 1.12 Å. For further details of data collection and processing, see Supplementary Table S1. In structures resulting from co-crystallization and in **1**-so-HEWL, the single HEWL chain has one ruthenium ion bound on the protein surface, while after the soaking procedure, two Ru ions can be identified in **2**-so-HEWL, **c**-so-HEWL, and **t**-so-HEWL (Figure 2). The overall conformations of the Ru–HEWL adducts are not significantly affected by the binding of metal, as evidenced by the absence of major changes in the main chain compared to the unmetalated HEWL form. For instance, the Cα root mean square (rms) deviations of the structures presented in this study from the HEWL–NAMI-A complex structure (PDBid: 4NY5) fall within the range of 0.14–0.18 (and 0.59–0.76 for all protein atoms). Regarding **2**-HL, when compared to the structure with PDBid: 3LN2, the Cα rms deviation is 0.229 (chain A) and 0.230 (chain B), while for all atoms, it is 0.872 (chain A) and 0.729 (chain B).

### 3.2 HEWL and HL ruthenium binding sites

The analysis of the difference Fourier maps clearly indicates strong positive peaks revealing the presence of Ru coordinated on the protein surface in the presented structures. In all determined structures, the electron density around the Ru center proves the absence of bulky organic ligands and dmso ligands. In **1**-co-HEWL and **2**-co-HEWL, a new coordination sphere composed of Arg14, His15, chloride ion, and water can be observed. The ruthenium ion is partially coordinated by the protein, while the rest of the Ru coordination sphere is fortified with small inorganic ligands (Supplementary Table S2). For 1-co-HEWL, two alternative conformations of Arg14 were refined. The Ru−NH1(Arg14A), Ru−NH2(Arg14B) distances are equal to 3.1 Å and 1.6 Å, respectively. The nitrogen NE2 of His15 is 2.7 Å away from the metal (Figure 2A). The coordination of ruthenium is completed by the chloride ion (distance to Ru of 3.1 Å). The coordination sphere visible on the Fourier map comprises four atoms. Given the 30% occupancy rate at which the Ru ion was refined, it is likely that the surrounding water molecules exhibit significant disorder. Consequently, the precise positions of water molecules completing the octahedral geometry of Ru remain undetermined. In structure **2**-co-HEWL, the distances from metal to Arg14 and His15 are different from those observed for the structures mentioned above; that is, Ru−NH1(Arg14) is 2.6 Å, Ru−NH2(Arg14) is 3.0 Å, and Ru−NE2(His15) is 2.8 Å (Figure 2B). The Ru coordination sphere, apart from the mentioned protein atoms, is composed of two water molecules (with distances of 2.2 Å and 2.7 Å from the metal) and a chloride ion that is 3.1 Å from the ruthenium center. In structure **2**-co-HEWL, similar to **1**-co-HEWL, only 30% of the protein fraction has a ruthenium binding site occupied by the metal. In **c**-co-HEWL and **t**-co-HEWL, relatively good resolutions revealed that no dmso molecule is coordinated to Ru and proved the existence of only small coordinating ions in the vicinity of the metal ion. Furthermore, in both structures, Ru adopts a distorted octahedral geometry, which is the most common geometry of ruthenium complexes in proteins (Vergara et al., 2013a; Messori and Merlino, 2017). The refinements resulted in high occupancy for both **c**-co-HEWL (occ. = 0.8) and **t**-co-HEWL (occ. = 0.8) structures and indicate a satisfactory degree of protein metalation. Interestingly, as was observed for **1**-co-HEWL and **2**-co-HEWL, only two residues were found to act as proteinogenic ligands that coordinate the Ru center in HEWL adducts formed with **c** and **t**. In **c**-co-HEWL, the metal ion is bound to the nitrogen NH1 atom of two alternative conformations observed for Arg14 and to the imidazole nitrogen NE2 of His15. The distances of Ru−NH1(Arg14B) and Ru−NE2(His15) are 2.0 Å and 2.4 Å, respectively (Figure 2C). The inorganic part of the ruthenium coordination sphere is decorated with three water molecules, positioned at distances of 2.1 Å, 2.3 Å, and 2.8 Å from the Ru atom. The sixth position is occupied by one chloride ion at a distance of 2.8 Å. Within **t**-co-HEWL, the coordination pattern involving protein residues shows similarity, particularly regarding the placement of the metal in the protein structure. The nitrogen NE2 atom of His15 was found at a distance of 2.4 Å from Ru. Additionally, two alternative conformations of Arg14 coordinate the metal: Ru−NH1(Arg14A), Ru−NH1(Arg14B) with distances equal to 2.7 Å and 2.1 Å, respectively (Figure 2D). The octahedral geometry of Ru is completed by two water molecules (2.1 Å and 2.2 Å from the metal center) and one chloride ion located 3.1 Å from the Ru cation.

Different preferences for the coordination of Ru are observed for HEWL crystals soaked with studied complexes. Surprisingly, soaking procedures led to the formation of adducts with metal ions mostly coordinated to Asp101 (Supplementary Table S2). In the case of 1-so-HEWL, the well-defined electron density centered at 2.3 Å from OD2 of Asp101 was further examined by anomalous mapping that supported the assignment of Ru ion (occ. = 0.63). Three solvent molecules could also be modeled around the metal ion (Figure 2F) at distances of 2.1 Å, 2.3 Å, and 2.3 Å, providing a distorted octahedral arrangement for Ru as previously observed. Two Ru ions could be identified in structure 2-so-HEWL: Ru1 (occ. = 0.17) at 2.7 Å from OD1(Asp101) and Ru2 (occ. = 0.31) at 2.2 Å from OD2(Asp101) (Figure 2G). The coordination sphere of Ru1 is composed of two water molecules in distances of 2.2 Å and 2.3 Å, while for the Ru2 with five water molecules, the distances are 2.2 Å, 2.2 Å, 2.4 Å, 2.4 Å, and 2.6 Å. The coordination of ruthenium ions in **c**-so-HEWL and **t**-so-HEWL also engages position Asp101. In structure **c**-so-HEWL, the distance from metal ions to aspartic acid Ru1−OD2(Asp101) is 2.3 Å, and in Ru2−OD1(Asp), the distance is 2.5 Å (Figure 2H) with occupancy of 0.82 and 0.44, respectively. Only two water molecules are identified close to the Ru1 at distances of 2.3 Å and 2.4 Å. The second ruthenium (Ru2) was coordinated with three water molecules identified at distances of 2.0 Å, 2.2 Å, and 2.4 Å from the metal. As a result of soaking HEWL with **t**, the adduct was formed by the coordination of two Ru ions, Ru1 (occ. = 0.57) next to the His14 and Ru2 (occ. = 0.92) in the vicinity of the residue Asp101 Ru1 (Figure 2I). The observation is that after soaking, *cis*-[RuCl_2_(dmso)_4_] and *trans*-[RuCl_2_(dmso)_4_] form different adducts that can be explained by their structures. In *cis*-Ru, the three dmso molecules are bonded through the sulfur in a facial configuration, while the fourth is O-bonded (Figure 1), whereas, in *trans*-Ru, all four dmso molecules are S-bonded in the equatorial plane (Figure 1). Those complexes exhibit different behavior in water, and consequently, *cis*-[RuCl_2_(dmso)_4_] proved to be more active in biological evaluation. The coordination geometry of ruthenium in determined structures can be described as a partially or fully formed distorted octahedral arrangement, which is the most common geometry for ruthenium complexes in biomolecules. The first coordination sphere comprises various atoms: nitrogen and oxygen atoms from the amino acids (Arg14, His15, and Asp101), oxygen atoms from water molecules, and chlorides (Supplementary Figure S2). The number of atoms present in the coordination sphere of Ru does not correlate with the method of adduct formation, ruthenium charge, or refined Ru occupancy, which suggests the significant impact of complex performance in aqueous environments on the coordination patterns observed in ruthenium complexes.

Our results support the idea that in ruthenium complexes that are analogs to NAMI-A, the Ru ion loses the N-heterocyclic ligand and dmso upon protein binding. Both His15 and Asp101 are the most important residues for ruthenium binding in HEWL. Notably, Asp101 is the primary binding site. Subsequently, coordination to His15, which mimics the N-heterocyclic ligand, is observed as a result of the interaction between Ru and the polypeptide chain of HEWL. Considering different charges of the ruthenium ion within the studied complexes (Ru^3+^ in **1** and **2**; Ru^2+^ in **c** and **t**), it is evident that the Ru^2+^ complexes form more stable adducts, as indicated by their noticeably higher occupancy and completed metal coordination. Additionally, the presence of two Ru ions per HEWL molecule after soaking, compared to a single Ru ion per molecule after co-crystallization, suggests that not only the solubility and hydrolysis pathway of ruthenium complex but also the hydrolysis time scale significantly influences the metal coordination to the protein. This finding is critical for the biomedical application of NAMI-A analogs. Furthermore, our results indicate that even a small change in Cl^−^ concentration (higher in co-crystallization and lower in soaking experiments) influences the composition of the metal coordination sphere. Chloride ligands are present in the Ru coordination sphere in all -co-HEWL structures, while in -so-HEWL, only water molecules surround the ruthenium. Two additional aspects of the investigation of HEWL and ruthenium complex interactions should be underlined: the necessity for detailed characterization of the metal compound under biologically relevant conditions and awareness of the tendency to form polynuclear Ru species, which can limit the concentration of free metal ions. Finally, the binding of Ru ions and their complexes to HEWL can be altered by the electrostatic potential surface (see Figure 2J). Charges on the protein surface can influence the local molecular arrangement between the ruthenium complex and amino acids. Consequently, to enhance the selectivity of Ru complexes, factors such as charge, size, and the number of ions participating in coordination to the protein should be considered HL ruthenium binding sites.

The **2**-HL structure presents the dimeric form in which only one molecule of the homodimer interacts with Ru after co-crystallization with **2** (Figure 2E). As observed in previous structures, the electron density around the Ru centers clearly indicates the absence of N-heterocyclic or dmso ligands. Interestingly, ruthenium ions were found only in the vicinity of chain B of the HL dimer. The first site with an occupancy of 0.76 is located on the protein surface, but no atoms are observed at a distance less than 3.0 Å from Ru1. The side chains of Arg107 (3.6 Å to Ru1), Arg113 (3.1 Å and 3.6 Å to Ru1), and Gln117 (3.4 Å to Ru1) surround the ruthenium ion, along with water molecules at distances of 3.2 Å and 3.5 Å. The fact that the same place in chain A is not occupied by the metal has a structural explanation. In chain A, the conformations of the corresponding arginine residues (107 and 113) are different and probably do not create an appropriate environment for the ruthenium ion. The second ruthenium localization is also not fully occupied (occ. = 0.69); the metal ion Ru2 is surrounded by Gln127 (Ru2-N_main_chain_ 3.15 Å), Gln129 (Ru2-N_main_chain_ 4.05 Å), and water molecules (2.4 Å and 3.0 Å to Ru2). See Supplementary Figure S3 for a comparison with the electron density map close to Arg107 and Gln127 occupied by the water molecules in the native and by the metal ions in ruthenated HL. Considering that in the refined HL structure, the atoms proximate to both Ru sites are notably distant from the metal ions, except for a single water molecule, the nature of the interactions facilitating ruthenium “binding” to this protein remains a puzzle. We can only speculate that after the aquation process, ruthenium ions still tend to reside in the environment created by the protein solvent shell; however, no strong interaction with the protein atoms can be observed. Because the HL structure was solved with a maximum resolution of 2.49 Å, the identification of water molecules around the ruthenium ion can be hindered. Comparable findings, which show that ruthenium ions without covalent bonds with protein/nucleic acids can be found in the crystal, were previously reported in the structure of the RNA duplex fragment where Ru is bound inside the major groove and interacts with the Hoogsteen site of guanines (PDBid: 1O3Z) or in HEWL crystals after crystallization with Ru (arene) complex where the metal site was refined 5 Å from any protein atom (PDBid: 5KJ9) (Hanif et al., 2016).

### 3.3 Comparison of HEWL and HL ruthenated crystal structures

In all cases, the metal ion is coordinated on the surface of the protein and does not interact with any residue of the active site of lysozyme. Although HEWL and HL share 56% of their sequence identity, the sites occupied by the ruthenium ions are different. Because position 15 (histidine in HEWL) is represented in the HL sequence by leucine, which has a lower coordinating property, no ruthenium ion was found at that site in the **2**-HL structure. The same observation can be made for position Ser100 in HL, which corresponds to Asp101 in HEWL. Based on data from the literature, other common ruthenium binding sites in HEWL, such as Asp18, Asp53, and Asp120, which have their equivalents in the HL structure, might be considered for metalation. Nevertheless, complexes **1, 2,** as well as **c**, and **t**, did not lead to the formation of Ru–HL adducts by coordination of ruthenium to metalation sites located close to Asp18, Asp53, Asp101, or Asp120.

When comparing HL to HEWL, the metal binding site located close to the C-terminus of HL (Gln126–Cys128) resembles the one observed at the C-terminal of HEWL close to Leu129 (PDBid: 5OB6) and reported by Pontillo et al. (2017); however, the exact positions and their nature are different. The second HL metalation site located close to Arg107 and Arg113 has not been seen in any HEWL structure reported so far. Our findings reveal that metal-based drugs acting as prodrugs may also form adducts without a net preference for selected side chains such as His or Asp. The structures of HEWL and HL after ruthenation presented here demonstrate that the formation of metal–protein adducts can influence the protein molecule.

### 3.4 HEWL-bound Ru fraction quantification in aqueous solution

In order to verify the degree of interaction of **1** and **2** with HEWL in solution, both complexes were incubated with this protein for 24 h at 37°C in a buffered solution at pH 4.5, the same as used for crystallization. After ultrafiltration, the protein samples were analyzed by ICP-MS to determine Ru content. Both **1** and **2** ruthenated HEWL in solution (Table 1), and no more than one equivalent of Ru was bound to HEWL, which is consistent with the crystallographic studies. The formed adducts were stable enough to be separated from free Ru species, suggesting irreversible bond formation under the experimental conditions employed. This assumption is further supported by the elucidation of the first coordination sphere for Ru ion in adducts with HEWL based on the crystal structure, where at least two donor atoms come from the side chain of amino acids. Comparable studies were conducted at pH 7.4 to approach physiological conditions, indicating that the rise in pH had a minimal impact on the binding of Ru complexes to HEWL (Table 1).

**TABLE 1:** Ru fraction [mol/mol (protein)] determined by ICP-MS in ruthenated HEWL produced after incubation of **1** or **2** with HEWL at a 20:1 M ratio for 24 h at 37°C followed by extensive ultrafiltration to remove unbound Ru species.

Metal complex	pH	Ru/HEWL
[HIsq][*trans*-RuCl_4_(dmso)(Isq)]	4.5	0.88 ± 0.07
	7.4	0.83 ± 0.08
[H_2_Ind][*trans*-RuCl_4_(dmso)(HInd)]	4.5	0.62 ± 0.18
	7.4	1.82 ± 0.19

### 3.5 Hydrolytic behavior of 1 and 2 under acidic conditions

**FIGURE 3:**
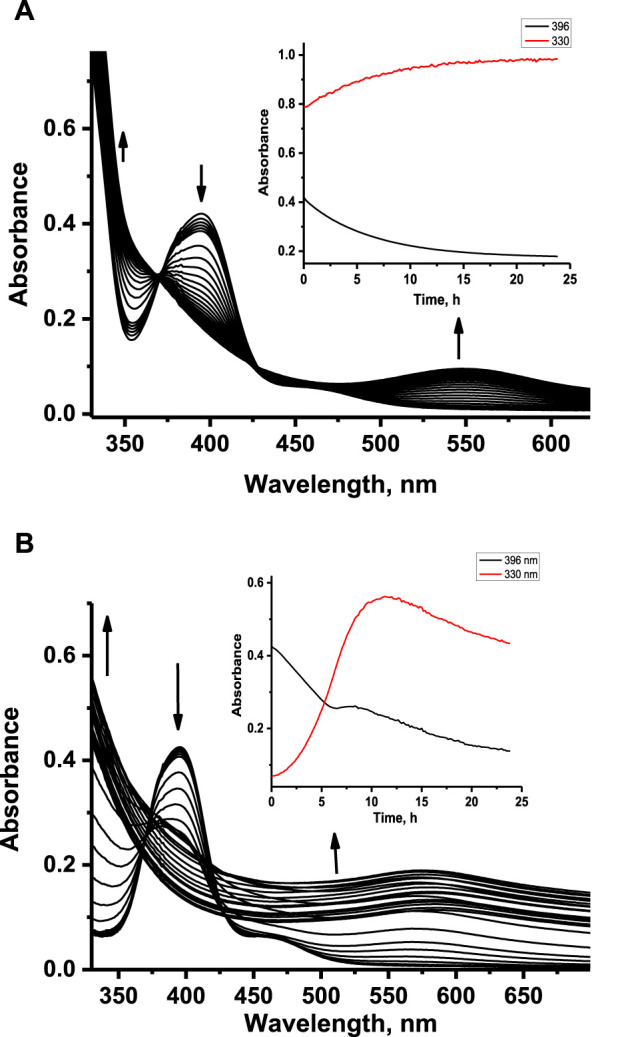
Absorbance changes registered upon aquation of ruthenium complexes **1 (A)** and **2 (B)** at pH 4.5. Insets: kinetic traces at 330 nm and 396 nm registered after 24 h aquation. [Ru] = 100 μM, [acetate buffer] = 0.05 M, [NaCl] = 0.2 M, 37°C.

It is well known that NAMI-A-type complexes undergo hydrolysis in aqueous solution with kinetics strongly dependent on pH. Because co-crystallization of ruthenium complexes **1** and **2** with HEWL required acidic pH (pH 4.5), the hydrolytic stability of these complexes was checked at the same pH conditions and compared with analogous studies under physiological-like conditions. The stability of ruthenium complexes at pH 4.5 was monitored spectrophotometrically. Freshly dissolved water solutions of **1** and **2** were rapidly mixed with acetate buffer and incubated at 37°C with concomitant registration of UV-Vis spectra at defined time intervals for 24 h (Figure 3). The registered spectral changes indicated the multiple-step nature of the aquation process, which is also confirmed by the lack of isosbestic points. In the first few hours of aquation, spectral changes for both complexes looked similar; namely, the disappearance of the Cl-to-Ru charge transfer band at 396 nm was observed with a simultaneous increase of the absorbance at a shorter wavelength. The darkening of the solutions after a longer period of time can be attributed to the formation of polynuclear Ru species. At pH 4.5, the disappearance of the charge transfer band of **1** and **2** at 396 nm takes approximately 20 h at 37°C. In contrast, at pH 7.4, this process is significantly faster and takes approximately 7 min (Oszajca et al., 2017). This observation agrees with the kinetics of the first aquation process of NAMI-A at acidic and physiological pH (Bouma et al., 2002; Bacac et al., 2004; Oszajca et al., 2014). Much slower aquation processes at pH 4.5 than physiological pH confirm that **1** and **2** are much more stable at acidic pH. The longer aquation process leads to increased absorbance at almost the entire registered UV-Vis range, leading to the formation of insoluble polynuclear Ru species.

**FIGURE 4:**
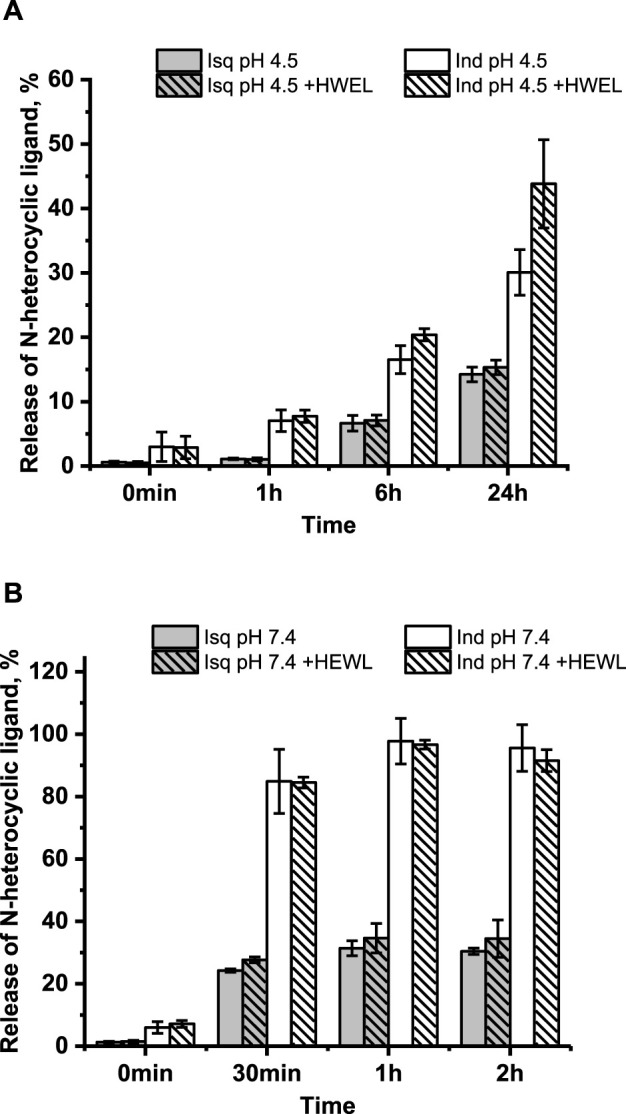
Time-dependent/resolved release of the N-heterocyclic ligand calculated using calibration curves (Supplementary Figure S5) at pH 4.5 without HEWL and in the presence of HEWL **(A)**; pH 7.4 without HEWL and in the presence of HEWL **(B)**. Experimental conditions: [Ru] = 10 μM, [HEWL] = 10 μM, [Tris buffer] = 0.1 M (pH 7.4), [NaCl] = 0.2 M; [acetate buffer] = 0.05 M (pH 4.5), [NaCl] = 0.2 M; 37°C, λ_ex_ = 295 nm, λ_em_ = 350 nm.

Our previous studies revealed that **1** and **2** release their N-heterocyclic ligand at physiologically similar pH (Oszajca et al., 2017); therefore, we decided to investigate how this process proceeds under acidic conditions. The time-resolved quantitative liberation of Ind or Isq was determined by HPLC technique with fluorescence detection because free indazole (Ind) and isoquinoline (Isq) exhibit significant fluorescence, whereas the fluorescence of coordinated N-heterocyclic ligands is completely quenched by the ruthenium center (Supplementary Figure S4). The amount of ligand released was calculated on the basis of the calibration curve prepared using authentic samples (Supplementary Figure S5). The release of Ind from **2** achieves approximately 30% after 24 h of incubation at pH 4.5, 37°C, while only approximately 15% of Isq abandons the coordination sphere of **1** (Figure 4A). This is much less than at pH 7.4, where Ind release from **2** is completed within 1 h, while Isq release from **1** achieves 30% and stays at this level for a longer incubation time (Figure 4B). Liberation of Ind or Isq ligands from the studied ruthenium complexes is in contrast to NAMI-A, for which no release of imidazole from the complex was observed under similar conditions (Bacac et al., 2004; Brindell et al., 2008). Analogous studies were performed for these complexes in the presence of equimolar protein concentration to determine if the presence of HEWL affects the stability of **1** and **2**. The results presented in Figure 4 confirm the lack of a substantial influence of HEWL on the release of N-heterocyclic ligands from the complexes. This suggests that N-heterocyclic ligands do not release as a result of the interaction with proteins, but rather, the dissociation of these ligands is required to make coordination bonds with the side chain of amino acid residues of the lysozyme. The elevated level of the free N-heterocyclic ligand was only observed after 24 h (at pH 4.5) when the increase in the coordination binding mode over the non-covalent mode was expected. For pH 7.4, the release of N-heterocyclic ligands was approximately twice that at acidic conditions, which seems to be sufficient for interaction with lysozyme.

**FIGURE 5:**
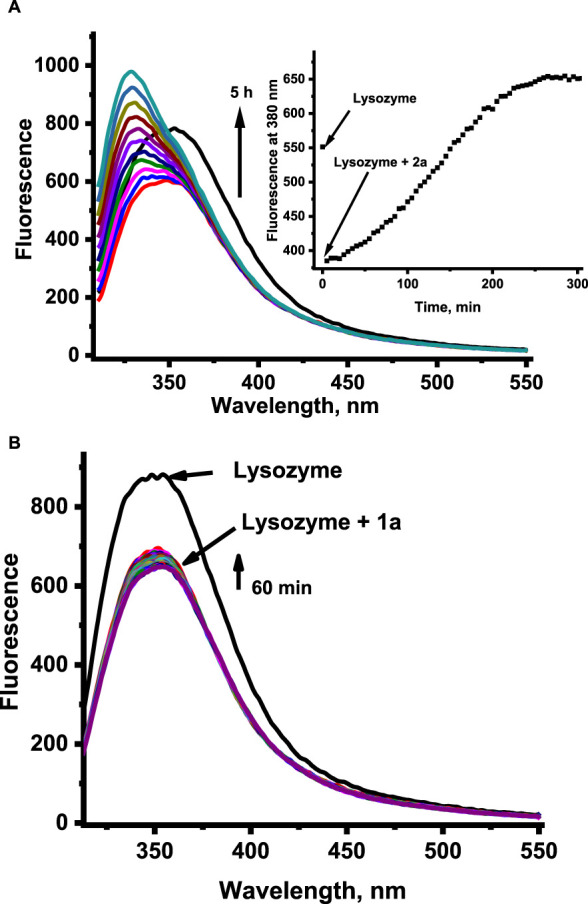
Fluorescence changes related to the liberation of the N-heterocyclic ligand from the ruthenium complexes **2** (for clarity, the graph shows the spectral changes from the initial reaction time) **(A)** and **1 (B)** in the presence of HEWL at pH 4.5. [Ru] = 20 μM, [Lys] = 2 μM, [acetate buffer] = 0.05 M pH 4.5, [NaCl] = 0.2 M, 37°C, λ_ex_ = 295 nm.

Time-dependent fluorescence spectral changes registered at pH 4.5 and 37°C during progressive aquation of **1** and **2** revealed a strong increase in fluorescence intensity originating from the free N-heterocyclic ligand in the case of **2**, whereas only slight fluorescence growth was detected for **1** (data not shown). The release of the coordinated N-heterocyclic ligand from **1** and **2** was also studied in the presence of HEWL to see if the presence of protein affects this process. Time-dependent fluorescence spectral changes registered with and without HEWL did not show any pronounced effect of protein on fluorescent ligand liberation. Significant differences can be observed when comparing the data at pH 4.5 and 7.4, as acidic conditions appear to slow the liberation of N-heterocyclic ligands and diminish the number of released ligands (Figure 5 compared with Supplementary Figure S6; pH 7.4).

### 3.6 Interaction of 1 and 2 with HEWL—protein fluorescence quenching

**FIGURE 6:**
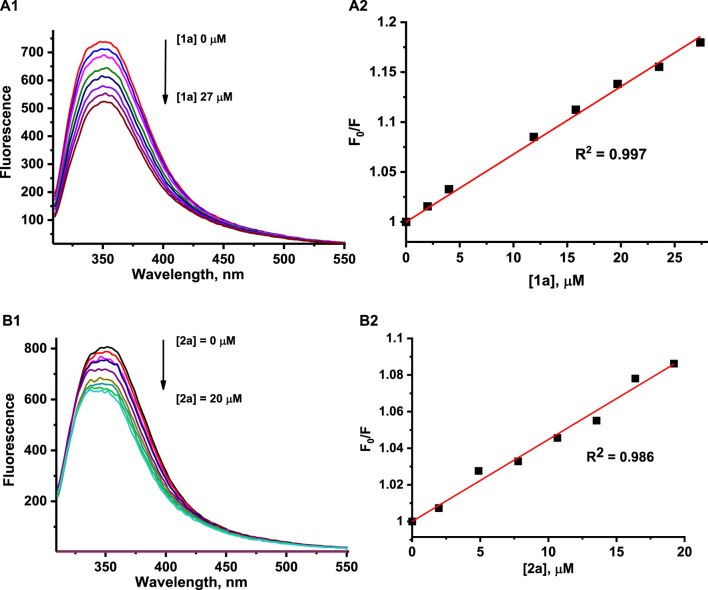
Fluorescence emission spectra for HEWL at pH 4.5 in the presence of increasing concentrations of **1a (A1)** and **2a (B1)**. The representative Stern–Volmer plot was determined at pH 4.5 by quenching the tryptophan fluorescence of HEWL by **1a (A2)** and **2a (B2)**. Experimental conditions: [HEWL] = 2 μM, [**1a**] = 0–27 μM, [**2a**] = 0–20 μM, [acetate buffer] = 0.05 M, [NaCl] = 0.2 M, 37°C, λ_ex_ = 295 nm, A2: λ_em_ = 350 nm (**1**), B2: λ_em_ = 380 nm (**2**).

HEWL possesses six Trp residues, and it is well known that fluorescence emission from Trp residues is very sensitive to changes in the local environment and can be used to monitor interactions with metal complexes, among others (Du et al., 2008; Montavon et al., 2009; Mazuryk et al., 2012, 2014). Such interaction results in quenching of protein fluorescence and allows the determination of the overall affinity constant. It is important to mention that the strong fluorescence of H_2_Ind^+^ and HIsq^+^ counter ions, which coincides with the fluorescence spectrum of HEWL while excited at 295 nm, as well as the release of fluorescent Ind and Isq ligands from the complex, generated serious difficulties while studying protein fluorescence quenching by **1** and **2**. Therefore, to be able to perform HEWL binding studies, the exchange of H_2_Ind^+^ and HIsq^+^ counter ions for Na^+^ was required, and all other solution studies were performed with application **1a** and **2a** complexes, that is, [Na^+^][*trans*-RuCl_4_(dmso)(Isq)] as **1a**, and [Na^+^][*trans*-RuCl_4_(dmso)(Hind)] as **2a**. To avoid the influence of the released Ind or Isq on the registered fluorescence intensities quenched by **1a** and **2a**, each sample of **1a** or **2a** was taken at an appropriate concentration from a fresh stock solution and added to individual samples of HEWL. The addition of **1a** or **2a** to HEWL at pH 4.5 resulted in a concentration-dependent decrease in the fluorescence intensity of HEWL (Figures 6A1, B1). A similar behavior was observed at pH 7.4 (Supplementary Figure S7).

The overall binding affinity constants for the reaction of the studied complexes with HEWL were determined by the application of a well-known Stern–Volmer equation: F_0_/F = 1 + *k*_q_•τ•[Q] = 1 + *K*_sv_•[Q], where F_0_ and F are the fluorescence intensities of HEWL measured in the absence and presence of quencher, respectively; *k*_q_ is the bimolecular quenching constant, τ_0_ is the average fluorescence lifetime, *K*_sv_ is the Stern–Volmer constant, and [Q] is the concentration of quencher (ruthenium complex) (Instrumentation for Fluorescence Spectroscopy, 2006). The measured intensities were corrected for the inner-filter effect and self-absorbance (see *Materials and methods*). Due to the complications described above, in the case of the **2a** complex, quenching constants were determined at 380 nm, where disruptions related to indazole liberation are minimized. The F_0_/F versus quencher concentration plots showed a linear dependence up to at least 10-fold the excess of **1a** or **2a** over the HEWL concentration at both pH values studied (Figures 6A2, B2; Supplementary Figure S8).

The determined Stern–Volmer constants, summarized in Table 2, are the mean values of three independent experiments. The overall binding affinity of the examined complexes to HEWL seems to be largely independent of both the nature of the N-heterocyclic ligand and pH (Table 2). All values of the determined Stern–Volmer constants are of a 10^3^ M^−1^ order of magnitude, suggesting a static mechanism of HEWL fluorescence quenching by the studied complexes. One of the supporting facts is that the fluorescence lifetime of HEWL (depending on pH and wavelength) is in the range of ca. 1.2–2.0 ns, giving the bimolecular quenching constants (*k*_q_) in the 10^12^ M^−1^ order of magnitude. The diffusion-controlled dynamic quenching constants typically are in the range of 10^10^ M^−1^; thus, larger values of *k*_q_ usually point toward some type of binding interaction.

**TABLE 2:** Comparison of Stern–Volmer constants determined at pH 4.5 and 7.4.

Quencher	*K*_SV_, mM^−1^, at 37°C	
	pH 4.5	pH 7.4
**1a**	6.8 ± 0.6	8.1 ± 0.1
**2a**	5.1 ± 0.6	4.5 ± 0.6

## 4 Discussion

As can be elucidated from the analysis of HEWL structures deposited to the Protein Data Bank, the ruthenium coordination can differ in terms of its localization and microenvironment. Ruthenium compounds like AziRu and NAMI-A serve as sources of the naked ruthenium ion that preferably interacts with histidine or aspartic acid residues located on the protein surface. AziRu tends to ruthenate HEWL (Vergara et al., 2013a) at His15, while co-crystallization with NAMI-A results in the metalation of Asp101 and Asp120 (Messori and Merlino, 2014). Protein–metal adducts were obtained with AziRu both by soaking and co-crystallization methods (two structures with Ru occupancy of 0.3 and 0.5; PDBid: 4J1A and 4J1B, respectively), and the complex was proved to be aquated (ligand exchange with *t*_*1/2*_ of approximately 65 h at pH 4.5) before coordination to His15 in the HEWL molecule (Vergara et al., 2013b). Interestingly, Messori and Merlino reported that for experiments with NAMI-A (PDBid: 4NY5), successful metal binding to Asp101 and Asp120 (both sites with occ. = 0.4) was obtained exclusively through co-crystallization, and also in this case, detachment of all ligands from the Ru(III) center was observed. Recent investigations by Chiniadis et al. on HEWL proved that HEWL can be effectively soaked with NAMI-A. They documented the initial coordination of the entire NAMI-A molecule to a “recognition site” at intervals of 1.5 h, 8 h, and 26 h, followed by a gradual disintegration of the three bound complexes (Ru ions occupancy ranging from 0.34 to 0.82). Notably, after 98 h, the researchers observed one Ru ion bonded to a known site near His15, which they identified as the “ruthenation site” (Chiniadis et al., 2021). Structural characterization was also undertaken for complexes arising from the interaction of ruthenium complexes with various proteins, extending beyond HEWL. Adduct of NAMI-A with human H-chain ferritin obtained by a soaking procedure reveals the selective binding of a single Ru ion to His105 (Ciambellotti et al., 2018) and the release of all Ru ligands. His105 is present in two different conformations, in which ruthenium occupies two alternative positions (Ru ion occupancy equal to 0.25 and 0.35; PDBid: 6FTV). On the other hand, NAMI-A in complex with human carbonic anhydrase II shows coordination of Ru ion to Asn62 and His64 (occ. = 0.8; PDBid: 3M1J) with simultaneous loss of all RuIII ligands upon protein binding (Casini et al., 2010). Soaking of RNase A crystals with AziRu results in protein metalation at His105 or His119 (occ. = 0.8; PDBid: 4L55) and the absence of primarily coordinated Ru ligands (Vergara et al., 2013b). Human serum albumin ruthenated by KP1019 showed two distinctive Ru binding sites. In site I, Ru is coordinated by His146, whereas, in site II, the Ru ion is bound to His242. In both Ru centers, occupancy of ∼0.5 (PDBid: 5IFO) indicates a similar degree of Ru affinity and protein metalation, resulting in the concomitant release of all other original Ru ligands (Bijelic et al., 2016). An adduct of proteinase K with RuTE formed by soaking crystals in excess RuTE-Cl ([RuII(1,4,7-trithiacyclononane)(ethane-1,2-diamine)Cl]^+^) revealed Ru binding at the two calcium-binding sites by Asp200 in site I and Asp260 in site II (occ. = 0.54 and 0.39 respectively; PDBid: 6TXG) (Chiniadis et al., 2020). Interestingly, complex formation is conditioned by chloride ligand release from the coordination sphere of RuTE-Cl, while the other two chelating ligands remain in place.

In our studies, both the soaking and the co-crystallization methods for HL with Ru complexes (**1**, **c**, **t)** were not successful. This is not a single case of difficulties in the formation of adducts with lysozyme. Vergara et al. reported that the crystallization experiments for HEWL with KP1019 did not give positive results after either soaking pre-grown crystals or in the case of co-crystallization (Vergara et al., 2013b; 2013a). Consequently, there remains a gap in studies related to the structural analysis of protein interactions with important Ru-based anticancer compounds that are structurally similar. What can be concluded so far is that coordination of Ru ion *via* interaction with His15 appears to be the most frequent and was identified in many structures, for example, as a result of HEWL crystallization with the carbon monoxide-releasing molecule (CORM) *fac*-Ru(CO)_3_Cl (κ^2^-H_2_NCH_2_-CO_2_) (PDBid: 2XJW) (Seixas et al., 2015a) and with three other CORM-related complexes of type [Ru(CO)_3_Cl_2_L] where L is: N^3^-C_5_H_4_(CH_2_)_2_SO_3_Na (PDBid: 4UWN) (Seixas et al., 2015a), N^4^-C_5_H_4_(CH_2_)_2_SO_3_Na (PDBid: 4UWU) (Seixas et al., 2015a), or water-soluble phosphine PTA (1,3,5-triaza-7-phosphaadamantane) (PDBid: 4UWV) (Seixas et al., 2015b). All these complexes have a therapeutic activity attributed to their ability to deliver CO to biological targets. Interestingly, the ruthenium occupation factor (from 0.5 up to 1.0) at the His15 metalation site after HEWL crystallization with these types of complexes is higher than observed for NAMI-A analogs. Furthermore, a wide range of HEWL crystal structures indicate that Asp18, Asp53, Asp101, and Asp120 can also serve as a ligand for ruthenium ions or ruthenium complexes. Coordination to Asp18 can be seen in structures with mentioned *fac*-Ru(CO)_3_Cl (κ^2^-H_2_NCH_2_-CO_2_) (PDBid: 2XJW) (Santos-Silva et al., 2011), but also with [Ru(CO)_3_Cl_2_]_2_ (PDBid: 4W96) (Tabe et al., 2015). In particular, binding sites close to Asp18 and Asp5 are occupied by Ru complexes that maintain their CO ligands. The region of Asp53, Asp101, and Asp120 occupied by Ru was reported by Santos-Silva et al. (2011) after HEWL soaking with *fac*-Ru(CO)_3_Cl (κ^2^-H_2_NCH_2_-CO_2_) (PDBid: 2XJW) and by Seixas et al. (2015b) for HEWL soaked with [Ru(CO)_3_Cl_2_(N^3^-C_5_H_4_(CH_2_)_2_SO_3_Na)] (PDBid: 4UWN). The last identified Ru binding site close to Asp120 can be occupied both by the bare ruthenium ion (PDBid: 5OB7) (Pontillo et al., 2017) resulting from HEWL co-crystallization with *fac*-[Ru^II^(CO)_3_Cl_2_(N^3^-methyl-imidazole)], as well as by Ru with CO ligands after co-crystallization with *fac*-[Ru^II^(CO)_3_Cl_2_(N^3^-imidazole)] (PDBid: 5OB6) (Pontillo et al., 2017). Overall, it can be concluded that the preference for the particular HEWL metalation sites could be influenced by the initial structure of the ruthenium complexes and strongly depends on the stability of the predominant form of the complex in solution. Despite the increasing number of studies on these aspects, a comprehensive understanding of the preferences of ruthenium compounds for a specific protein binding site and the potential therapeutic effects of the ultimately formed adducts is still lacking.

Combining crystallographic and in-solution spectroscopic studies has provided complementary information on the interaction of two NAMI-A analogs in which the imidazole ligand was replaced by either isoquinoline (**1**) or indazole (**2**). Additionally, the crystal structure reported herein offers a detailed description of the **2**-HL adduct. In agreement with the previous investigations, crystallographic data confirm that the studied complexes bind to lysozyme after the liberation of most of their original ligands. In the case of the studied ruthenium complexes (**1** and **2**), dissociation occurs prior to binding, as supported by in-solution studies that show no increased dissociation of Isq or Ind in the presence of HEWL. This is further supported by the fact that **c** and **t** complexes, which do not contain N-heterocyclic ligands, target the same amino acid residues as **1** and **2**, as demonstrated in co-crystallization and soaking experiments. Both **1** and **2** form adducts via metalation sites similar to those already determined for the AziRu-HEWL (Vergara et al., 2013a) and NAMI-A-HEWL adducts (Chiniadis et al., 2021). Complexes investigated by us form adducts by utilization of two binding sites: one next to Asp101 (the primary coordination site observed after the soaking experiment) and the second involving Ru coordination to imidazole of His15 and to Arg14 (the final coordination site identified in the co-crystallization experiments). It is noteworthy that most X-ray crystallographic structures of Ru complexes with various proteins result from prolonged co-crystallization, often extending over several days (Russo Krauss et al., 2013). Identification of distinct HEWL adducts following two protocols (co-crystallization and soaking) for the formation of Ru adducts underscores the different coordination chemistry depending on time. In our co-crystallization experiments, we observed a single ruthenium ion binding to the final coordination site, whereas soaking experiments mostly revealed two ruthenium ions at the primary coordination site. Therefore, time adds another pivotal aspect in directing ruthenium ions to the specific coordination sites beyond the characteristics of the N-heterocyclic ligand, its hydrolytic behavior, bulkiness, steric attributes, and affinity for specific areas on the protein’s electrostatic surface.

A notable finding is that the final binding site comprises histidine residue, indicating a strong preference of the ruthenium ion for histidine through a mechanism where the ion replaces the N-heterocyclic ligand in NAMI-A. Further analysis shows that the occupancy rates at these sites vary between *cis*/*trans* and NAMI-A-type complexes. Experiments involving co-crystallization and soaking with *cis*-[RuCl_2_(dmso)_4_] and *trans*-[RuCl_2_(dmso)_4_] isomers demonstrate higher occupancy rates than complexes **1** and **2**. This observation highlights the crucial role that the chemical properties in aqueous environments play in adduct formation. Moreover, it was through soaking experiments that the unique chemical behavior of the complexes in water was proved. The stability of ruthenium complexes **1** and **2** in aqueous solutions correspond to the Ru1 occupancy rate of 0.63 in **1**-co-HEWL and to the significantly lower Ru1 and Ru2 occupancy rates of 0.17 and 0.31, respectively, observed in **2**-co-HEWL. In addition, lower occupancies are also observed for ruthenium ions in **c**-co-HEWL (0.44 and 0.82) compared to **t**-co-HEWL (0.57 and 0.92).

The hydrolysis of **c** and **t** complexes proceeds as follows: once *cis*-[RuCl_2_(dmso)_4_] is dissolved in water, it immediately releases the O-bonded dimethyl sulfoxide molecule while the dissolved *trans*-[RuCl_2_(dmso)_4_] isomer releases two dimethyl sulfoxide molecules (Alessio et al., 1988; Brindell et al., 2007). This initial step is followed by the slow dissociation of a Cl^−^ anion, leading to the formation of cationic species for both isomers. Such behaviors influence occupancy levels, evidence that aligns with the biological evaluations demonstrating that the *trans*-[RuCl_2_(dmso)_4_] exhibits higher antineoplastic activity (Sava et al., 1984), antitumor properties (Sava et al., 1989), and antiproliferative activity (Brindell et al., 2005) than the *cis*-[RuCl_2_(dmso)_4_] isomer. Finally, the similarity in the Ru coordination sphere across the HEWL adducts after co-crystallization or soaking with **c** and **t** (Ru^2+^), **1** and **2** (Ru^3+^) suggests that the metal’s ion charge does not predominantly dictate the binding site specificity of the ruthenium center but instead influences the level of complexation. Our findings emphasize the influence of the rate and pathway of ligand dissociation from the metal center in determining the final binding site of the ruthenium ion on the protein. However, the interplay of the aforementioned factors implies that our experimental techniques may only capture a fragment of the Ru–protein interaction spectrum, leaving the complete landscape yet to be fully explored.

## 5 Conclusion

The investigations presented here shed light on the interaction between two NAMI-A analogs, complexes **1** and **2**, and lysozyme (HEWL and HL), providing valuable insights into the potential therapeutic applications of ruthenium complexes. Through crystallographic analysis and solution-based studies, we delineated the coordination patterns of ruthenium ions on the protein surface, elucidating the factors influencing metallodrug–protein interactions. The kinetics and mechanistic pathways of ligand dissociation from the ruthenium center dictate the specific binding site on the protein. Therefore, the inherent lability of ligands in NAMI-A-type ruthenium complexes presents challenges in controlling their hydrolytic processes under physiological conditions. This unregulated hydrolysis significantly impacts their protein binding targets and therapeutic efficacy. One potential approach to mitigate the unpredictable hydrolytic behavior involves incorporating chelate ligands, such as polypyridyl derivatives, into the ruthenium center. Nevertheless, the exploration of non-coordinative binding and its role in protein interactions remains an open area of research. Our findings highlight the importance of understanding how the nature of N-heterocyclic ligands and hydrolytic behavior affect the binding of ruthenium complexes to proteins. Despite the challenges posed by the hydrolysis pathway and the nature of metal coordination, our results demonstrate the stability of the formed adducts, suggesting irreversible bond formation under experimental conditions. Moreover, we observed that the presence of proteins did not substantially alter the stability of the complexes or the release of N-heterocyclic ligands. The comparison with other metalated HEWL structures revealed the diverse coordination preferences of ruthenium compounds, emphasizing the need for a comprehensive understanding of the interactions between ruthenium complexes and proteins. Our study provides valuable insights into the coordination and stability of ruthenium complexes with HEWL and HL. We believe that our results essentially support previous studies and provide a deeper insight into the factors determining the interaction of NAMI-A-type Ru complexes with proteins. A thorough understanding of protein–metal recognition mechanisms holds promise for advancing the biochemistry of metallodrugs. This knowledge contributes not only to the foundational understanding of metal-related disease processes but also to the development and identification of novel drug targets and to the design of new drugs with desirable characteristics (e.g., targeted cancer treatment and bioimaging applications) (Riccardi et al., 2017; Xu et al., 2022; Nyong-Bassey et al., 2023; Singh et al., 2023). In the contemporary field of theranostics, ruthenium complexes stand out for their dual diagnostic and therapeutic capabilities, underscoring their growing significance in this innovative field.

## Data Availability

The datasets presented in this study can be found in online repositories. The names of the repository/repositories and accession number(s) can be found at: http://www.wwpdb.org/, 5LVG; http://www.wwpdb.org/, 5LVH; http://www.wwpdb.org/, 5LVI; http://www.wwpdb.org/, 5LVJ; http://www.wwpdb.org/, 5LVK; http://www.wwpdb.org/, 8RNV; http://www.wwpdb.org/, 8RNW; http://www.wwpdb.org/, 8RNX; http://www.wwpdb.org/, 8RNY.
