# Supplementary material for: Exploring the coordination chemistry of ruthenium complexes with lysozymes: structural and in-solution studies
Source: Front Chem. 2024 Apr 4;12:1371637. doi: 10.3389/fchem.2024.1371637 (PMC11024358; doi:10.3389/fchem.2024.1371637)
Supplement: Supplementary file 1 [file DataSheet1.pdf]

Supplementary Material

1 Supplementary Figures and Tables

1.1 Supplementary Figures

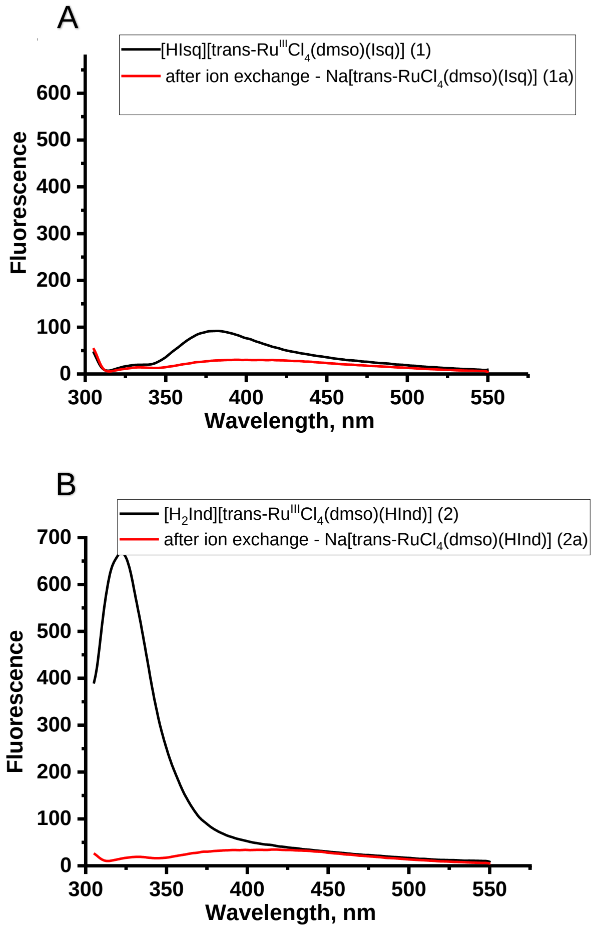

Supplementary Figure 1. Fluorescence spectra registered for complex **1** (A) and **2** (B) before ion exchange and after ion exchange procedure. [**1**/**2**] = 3 μM, [**1a**/**2a**] = 30 μM, [acetate buffer] = 0,05 M, pH 4.5, [NaCl] = 0.2 M, 37 °C, λ_{ex} = 295 nm, ex. slit = 15, em. slit = 7.5.

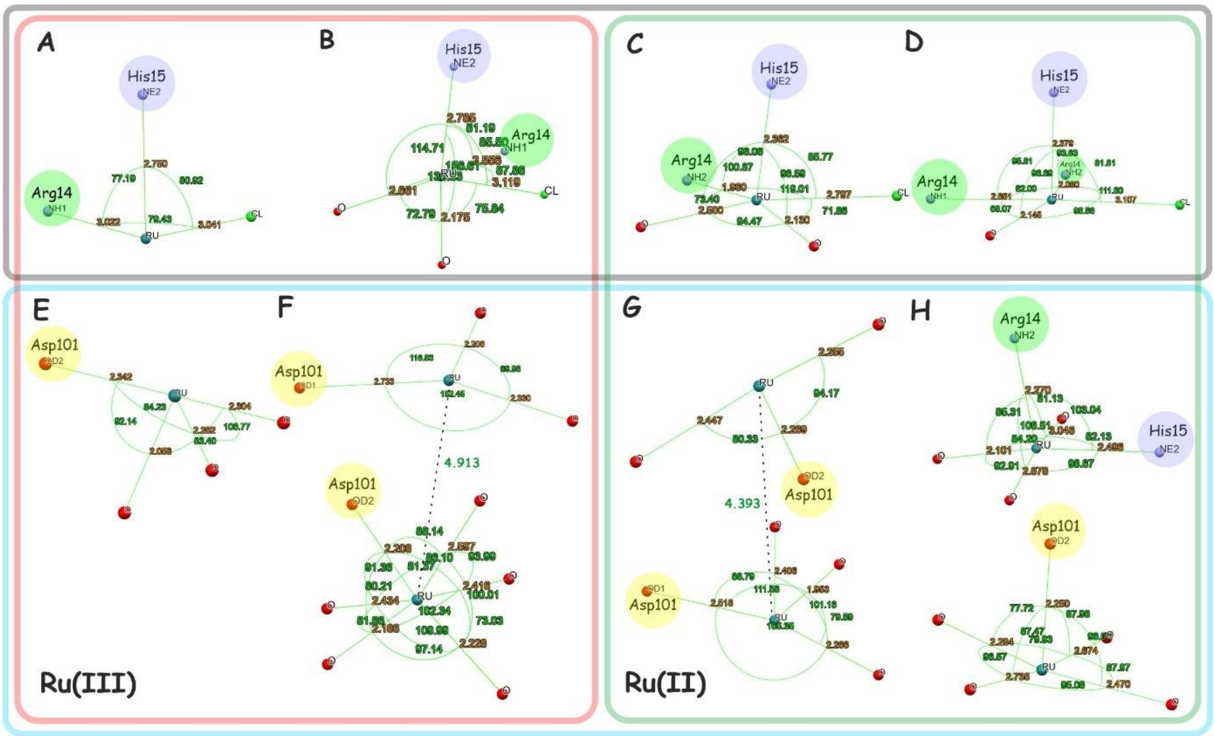

Supplementary Figure 2. Coordination of Ru in HEWL structures: (A) **1**-co-HEWL (PDBid: 5LVI), (B) **2**-co-HEWL (PDBid: 5LVJ), (C) **c**-co-HEWL (PDBid: 5LVG), (D) **t**-co-HEWL (PDBid: 5LVH), (E) **1**-so-HEWL (PDBid: 8RNX), (F) **2**-co-HEWL (PDBid: 8RNY), (G) **c**-co-HEWL (PDBid: 8RNV), (H) **t**-co-HEWL (PDBid:8RNW), co-crystallization (light grey frame), soaking (light blue frame); distances are given in Å, angles in degrees.

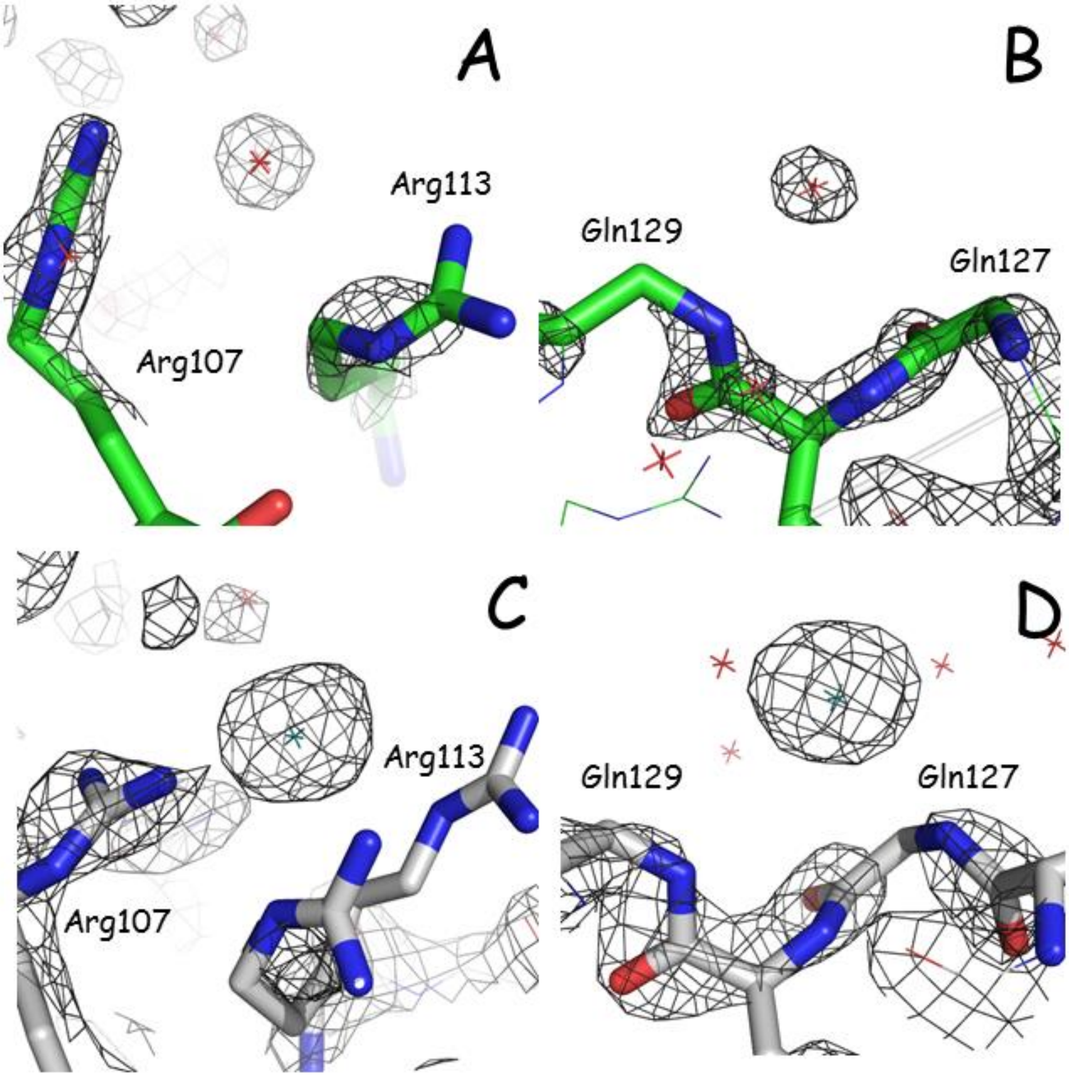

Supplementary Figure 3. Electron density maps close to Arg107 (A, C) and Gln127 (B, D) in native HL (PDBid: 3LN2) and ruthenated HL, respectively. In native HL structure water molecules can be found at the sites occupied by metal ions in ruthenated HL structure.

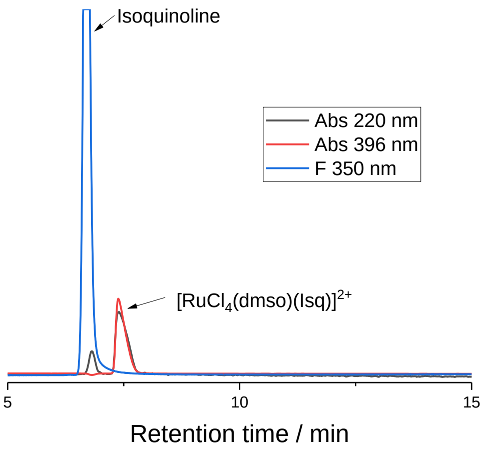

Supplementary Figure 4. Absorption (red and black line) and fluorescence (blue line) elution profiles registered for [HIsq][*trans*-$Ru^{III}Cl_4$(dmso)(Isq)] (**1**). The experimental condition are the same as depicted in Experimental section.

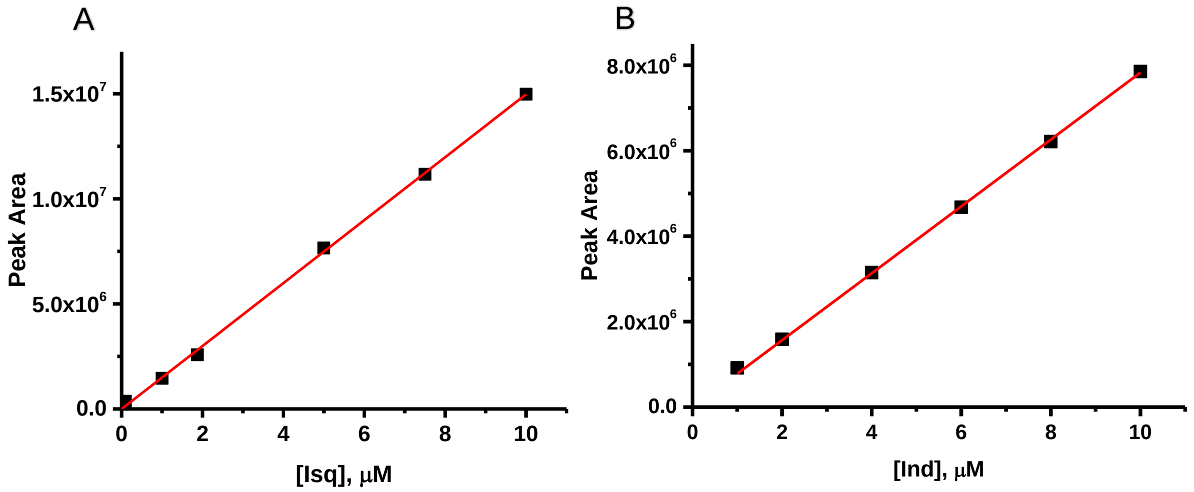

Supplementary Figure 5. Representative calibration curves for isoqinoline (A) and indazole (B). As a measure of N-heterocyclic concentration, the peak area was chosen, λ_{ex} = 295 nm, λ_{em} = 350 nm.

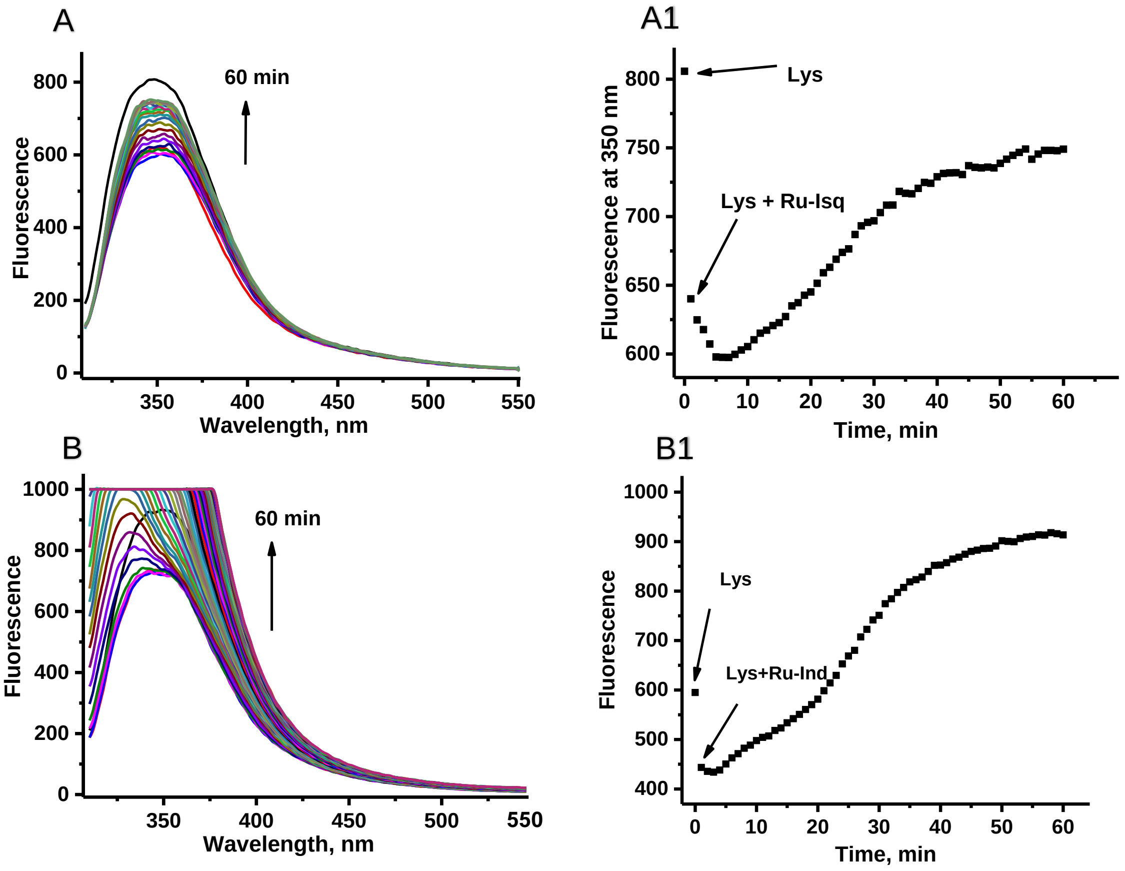

Supplementary Figure 6. Fluorescence changes after addition of excess of ruthenium complexes **1** (A and A1) and **2** (B and B1) to lysozyme at pH 7.4. [Ru] = 20 μM, [Lys] = 2 μM, [Tris buffer] = 0.1 M, pH 7.4, 37 °C, λ_{ex} = 295 nm.

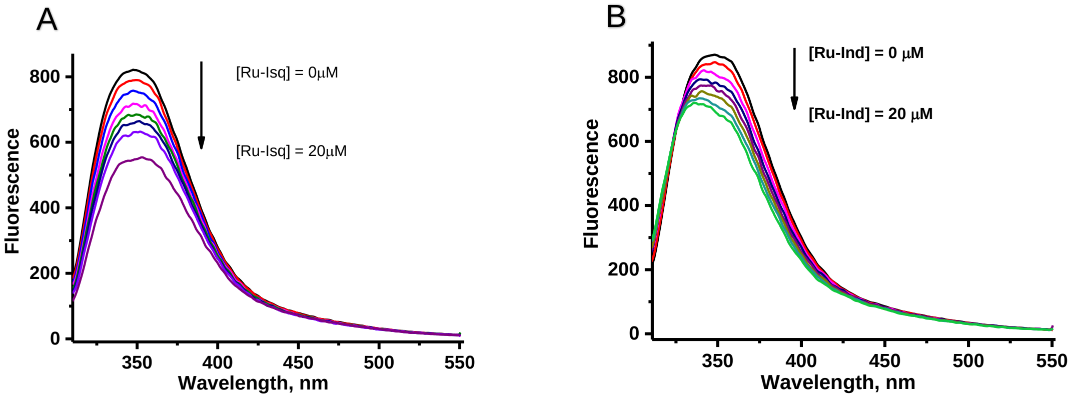

Supplementary Figure 7. Fluorescence emission spectra for lysozyme at pH 7.4 in presence of the increasing concentration of **1** (A) and **2** (B). Experimental conditions: [Lysozyme] = 2 μM; [**1a**] = 0 – 20 μM; [**2a**] = 0 – 20 μM, [Tris buffer] = 0.1 M, [NaCl] = 0.2 M, 37 °C, λ_{ex} = 295 nm.

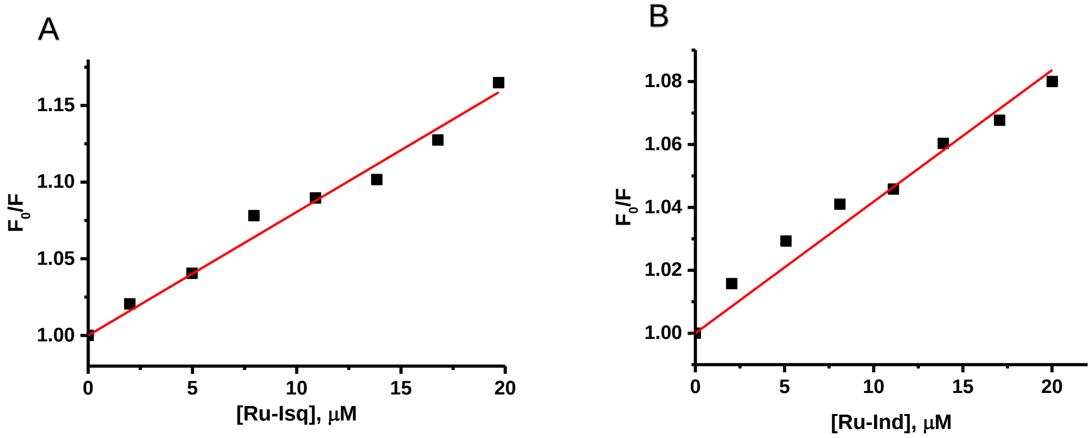

Supplementary Figure 8. The representative Stern-Volmer plot determined at pH 7.4 by quenching tryptophan fluorescence of lysozyme by **1** (A) and **2** (B). Experimental conditions: [Lysozyme] = 2 μM, [acetate buffer] = 0.05 M , [NaCl] = 0.2 M, 37 °C, λ_{ex} = 295 nm, λ_{em} = 350 nm (**1a**), λ_{em} = 380 nm (**2a**).

1.2 Supplementary Tables

Supplementary Table 1. Statistics of data collection and refinement. Values in parentheses are for the outer shell.

	CO-CRYSTALLIZATION					SOAKING			
	**c**-co-HEWL	**t**-co-HEWL	**1**-co-HEWL	**2**-co-HEWL	**2**-HL	**c**-so-HEWL	**t**-so-HEWL	**1**-so-HEWL	**2**-so-HEWL
PDBid	5LVG	5LVH	5LVI	5LVJ	5LVK	8RNV	8RNW	8RNX	8RNY
Max. resolution (Å)	2.00	1.50	1.49	1.50	2.49	1.08	1.12	1.25	1.02
Nr of reflections	58 202	112 340	112 526	110 309	54 634	613 041	558 486	405 185	651 223
Nr of unique reflections	8 212	18 815	18 827	18 789	11 854	48 247	44 798	32 398	57 608
Mosaicity (˚)	0.53	0.65	0.56	0.44	0.75	0.07	0.13	0.35	0.14
Resolution range (Å)	14.52-2.00 (2.00-2.05)	15.23-1.50 (1.50-1.52)	15.08-1.49 (1.49-1.52)	15.22-1.50 (1.50-1.52)	14.37-2.49 (2.00-2.06)	39.27-1.08 (1.10-1.08)	39.10-1.12 (1.14-1.12)	39.50-1.25 (1.27-1.25)	39.44-1.02 (1.04-1.02)
$R(I)_{merge}$	0.120 (0.254)	0.063 (0.193)	0.158 (0.410)	0.131 (0.250)	0.170 (0.263)	0.016 (0.154)	0.015 (0.216)	0.023 (0.208)	0.013 (0.217)
Completeness (%)	98.7 (100)	99.6 (99.3)	99.6 (94.9)	99.8 (97.2)	88.7 (82.4)	96.2 (76.9)	99.6 (96.1)	98.2 (74.8)	95.0 (65.7)
I/σ(I)	22.9 (6.6)	16.0 (1.6)	10.3 (0.6)	9.9 (0.7)	14.5 (1.2)	19.0(1.1)	20.4 (1.1)	11.4 (1.1)	18.7 (1.3)
$CC_{1/2}$	0.993 (0.978)	0.999 (0.567)	0.995 (0.517)	0.996 (0.533)	0.996 (0.543)	1.000 (0.569)	1.000 (0.550)	0.999 (0.633)	1.000 (0.673)
Space group	$P4_32_12$				$P2_12_12_1$	$P4_32_12$			
Unit-cell parameters									
a (Å)	78.20	77.68	77.48	77.61	42.04	78.54	78.21	78.99	78.88
b (Å)	78.20	77.68	77.48	77.61	64.10	78.54	78.21	78.99	78.88
c (Å)	37.19	37.19	37.16	37.21	111.31	37.11	37.19	37.00	37.03
Overall *B* factor from Wilson plot ($Å^2$)	13.5	15.1	15.3	15.1	10.6	18.3	19.9	24.0	14.6
Refinement resolution range (Å)	14.42-2.00	15.23-1.55	14.8-1.70	14.83-1.60	14.37-2.49	18.06-1.08	18.97-1.12	19.16-1.25	19.13-1.02
R (%)	15.77	17.10	16.26	17.31	18.77	15.43	15.55	22.19	12.94

R_{free} (%)	23.26	21.05	21.73	21.38	26.11	16.72	17.10	24.80	14.44
Average B-factor (Å^2)	15	18	18	19	12	15	17	18	12
Stereochemical restraints, r.m.s.									
Bond distance (Å)	0.016	0.019	0.019	0.019	0.049	0.005	0.005	0.006	0.006
Bond angles (°)	0.988	1.014	1.478	1.828	0.957	0.823	0.834	0.873	0.931
Core Ramachandran (%)	97.7	98.5	97.9	97.8	94.6	100	100	100	100

Supplementary Table 2. Details of the metalation sites in HEWL and HL ruthenated crystals (distances are given in Å).

	**CO-CRYSTALLIZATION**						**SOAKING**						
	**c**-co-HEWL	**t**-co-HEWL	**1**-co-HEWL	**2**-co-HEWL	**2**-HL		**c**-so-HEWL		**t**-so-HEWL		**1**-so-HEWL	**2**-so-HEWL	
Ru occupancy	0.8	0.8	0.3	0.3	0.76	0.69	0.82	0.44	0.57	0.92	0.63	0.17	0.31
Distance to protein atoms	Ru-His15<br>2.4	Ru-His15<br>2.4	Ru-His15<br>2.7	Ru-His15<br>2.8	Ru1-Arg113<br>3.1<br>3.6	Ru2-Gln127<br>3.15	Ru1-Asp101<br>2.3	Ru2-Asp101<br>2.5	Ru1-His15<br>2.5	Ru2-Asp101<br>2.3	Ru-Asp101<br>2.3	Ru1-Asp101<br>2.2	Ru2-Asp101<br>2.3
	Ru-Arg14<br>2.0	Ru-Arg14<br>2.7<br>2.1	Ru-Arg14<br>3.0	Ru-Arg14<br>2.6<br>3.0	Ru1-Arg107<br>3.6	Ru2-Gln129<br>4.0			Ru1-Arg14<br>2.3				
					Ru1-Gln117<br>3.4								
Distance to water molecules	2.1<br>2.3<br>2.8	2.1<br>2.2	2.3	2.2<br>2.7	3.2<br>3.5	2.4<br>3.0<br>3.8	2.3<br>2.4	2.0<br>2.3<br>2.4	2.1<br>2.9<br>3.0	2.3<br>2.5<br>2.7<br>2.7	2.1<br>2.3<br>2.3	2.2<br>2.3	2.2<br>2.3
Cl^-	2.8	3.1	3.0	3.1	-	-	-	-	-	-	-	-	
